# Necroptosis occurs in osteoblasts during tumor necrosis factor-α stimulation and caspase-8 inhibition

**DOI:** 10.1590/1414-431X20187844

**Published:** 2018-11-23

**Authors:** Guan Shi, Pu Jia, Hao Chen, Li Bao, Fei Feng, Hai Tang

**Affiliations:** Department of Orthopedics, Beijing Friendship Hospital, Capital Medical University, Beijing, China

**Keywords:** Necroptosis, Mouse osteoblast, Z-IETD-FMK, Necrostatin-1, TNF-α

## Abstract

Necroptosis is a regulated cell death mechanism. However, it is unknown whether necroptosis is involved in the death of tumor necrosis factor-α (TNF-α)-treated osteoblasts. Therefore, we conducted the study with TNF-α, Nec-1 (a specific inhibitor of necroptosis), and Z-IETD-FMK (a specific inhibitor of apoptosis) to determine whether necroptosis plays a role in the death of TNF-α-treated osteoblast cell line MC3T3-E1. Cell viability, cell death, and lactate dehydrogenase (LDH) release were assayed to evaluate cytotoxicity. Specific marker proteins receptor interacting protein kinase (RIPK3) and phosphorylated mixed lineage kinase domain-like protein (p-MLKL) for necroptosis, and cleaved caspase 3 for apoptosis were detected by western blot, and mRNA was measured by quantitative real-time polymerase chain reaction (qRT-PCR). We found that TNF-α inhibited cell proliferation in a dose- and time-dependent manner. Nec-1 plus Z-IETD-FMK restored cell viability and significantly decreased LDH release. In addition, TNF-α alone increased the cell population of AV+PI−, while Z-IETD-FMK caused a shift in the cell population from AV+PI− to AV+PI+. Furthermore, TNF-α significantly increased protein cleaved caspase 3. TNF-α plus Z-IETD-FMK significantly increased the proteins RIPK3 and MLKL phosphorylation in MC3T3-E1 cells, while the changes in mRNA levels of RIPK3, MLKL, and caspase 3 were not consistent with the changes in the corresponding protein expression levels. In conclusion, TNF-α induced preferentially apoptosis in osteoblast cell line and necroptosis played a decisive role when TNF-α-induced death was inhibited by the inhibitor of apoptosis. Combined treatment with Nec-1 and Z-IETD-FMK protected mouse osteoblasts from death induced by TNF-α.

## Introduction

Necroptosis is a non-caspase-dependent mechanism of regulated cell death (1). Its initiation, execution, and inhibition involve the expression and regulation of receptor interacting protein kinase 1 (RIPK1) and mixed lineage kinase domain-like protein (MLKL). In most cases, necroptosis is initiated by tumor necrosis factor-α (TNF-α) binding to TNF-α receptors. Nec-1 specifically inhibits necroptosis by interacting with RIPK1. Necroptotic cells are characterized by organelle swelling, disruption of cell membranes, cell disintegration, and the release of reactive oxygen species (2). This process may trigger innate and adaptive immune responses, which clear necroptotic cells through pinosome-mediated uptake.

Like necrosis and apoptosis, necroptosis is closely related to the occurrence, development, and prognosis of diseases, such as inflammation (3), ischemic injury (4), neurodegenerative diseases (5), and cancer (6). In addition, several studies have showed that Nec-1 can protect various cell lines from death and improve disease prognosis (7–9).

However, it is unknown whether necroptosis is involved in TNF-α-induced osteoblast death. If yes, then these cells could be protected by treatment with Nec-1, which could be an effective clinical strategy for treating orthopedic diseases caused by TNF-α. Therefore, we established a TNF-α-treated osteoblast model to determine whether necroptosis plays a role in TNF-α-induced mouse osteoblast cell death.

## Material and Methods

### Cell culture and chemicals

The MC3T3-E1 osteoblast cell line was provided by the Institute of Basic Medical Sciences (China). Cells were incubated in α-MEM medium (32571036; Gibco, USA) containing glutamine, 10% fetal bovine serum (10099-141; Gibco, Australia) and 1% penicillin/streptomycin (15140-122; Gibco, USA). Recombinant mouse TNF-α was purchased from Peprotech (AF-315-01A-20; USA). Nec-1 was purchased from MedChemExpress (4311-88-0; USA). Z-IETD-FMK, a specific inhibitor of caspase 8, was purchased from Selleck (S7314; USA). The primary antibodies used to detect p-MLKL (ab196436) and RIPK3 (ab56164) were purchased from Abcam (UK). The primary antibodies used to detect MLKL (37705S), cleaved caspase 3 (9661s), and caspase 3 (9662s) were obtained from Cell Signaling Technology (USA). β-actin (TA-09) and goat anti-mouse/rabbit IgG secondary antibodies were purchased from ZSGB-BIO (China).

### Cell viability

MC3T3-E1 cells were inoculated into 96-well culture dishes. When the cells grew to approximately 60% confluency, the culture medium was renewed, and the cells were treated with TNF-α (0, 5, 20, 50, or 100 ng/mL) for 12 and 24 h. Next, the medium was renewed, 100 µL of 10% CCK-8 (Dojindo, Japan) medium was added to each well, and cells were incubated at 37°C for 3 h. The absorbance was measured at a wavelength of 490 nm using a microplate reader (Model 680; Bio-Rad Laboratories, USA). The same method was applied to measure the absorbance of cells after treatment with inhibitors. Specifically, the cells were treated with Z-IETD-FMK (40 µM), Nec-1 (50 µM), or Z-IETD-FMK (40 µM) and Nec-1 (50 µM) for 1 h, after which TNF-α was added (0, 5, 20, 50, or 100 ng/mL) and the cells were incubated for another 24 h. Cell viability was calculated from a formula based on the absorbance according to the CCK8 manufacturer's instructions: Cell viability (%) = (A (dosing) - A (blank)) / (A (0 dosing) - A (blank)) × 100%, where A (dosing) is the absorbance of cells treated with CCK8 and drugs; A (blank) is the absorbance of medium and CCK8 solution without cells; and A (0 dosing) is the absorbance of cells treated with CCK8 but without drugs. The values on the y-axis indicate the ratio of the number of viable cells to the blank control group, which indirectly indicates the effect of the drugs on cell proliferation.

### Cell death assay

MC3T3-E1 cells were inoculated into 24-well culture dishes. Next, the cells were treated with Z-IETD-FMK (40 µM), Nec-1 (50 µM), or Z-IETD-FMK (40 µM) and Nec-1 (50 µM) for 1 h, after which they were treated with TNF-α (20 ng/mL) for an additional 12 or 24 h. Before analysis, the cells were washed twice with PBS and resuspended in binding buffer. Antibody staining was performed to detect Annexin-V-FITC (fluorescein isothiocyanate)/PI (propidium iodide) using an apoptosis kit according to the manufacturer's instructions (556547; BD Biosciences, USA). Briefly, 5 µL of Annexin-V-FITC and 5 µL of PI were added to each tube. The mixture was incubated in the dark at room temperature for 15 min. Cell death was analyzed by flow cytometry (FACS; BD Biosciences).

### LDH release assay

MC3T3-E1 cells were inoculated into 96-well culture dishes. When the cells reached approximately 60% confluency, the culture medium was removed and changed to 1% serum culture medium. After treatment for 6, 12, or 24 h, the dishes were centrifuged in a multiwell plate centrifuge at 400 *g* for 5 min at 25^o^C. Then, 120 μL of the supernatant from each well was transferred to a new 96-well plate and mixed with 60 μL of LDH test solution (Thermo Fisher Scientific, China) at 25°C for 30 min in the dark. The absorbance was then measured at 490 nm (Model 680; Bio-Rad Laboratories). The percentage of LDH that was released compared with the total LDH was calculated by subtracting the absorbance of the blank control from the measured absorbance for each group according to the following formula: (absorbance of drug-treated cell group – absorbance of sample control group) / (absorbance of sample maximum enzyme activity control group – sample control group absorbance) × 100.

### Western blot analysis

The cells were treated with Z-IETD-FMK (40 µM), Nec-1 (50 µM), or Z-IETD-FMK (40 µM) and Nec-1 (50 µM) for 1 h, after which they were treated with TNF-α (20 ng/mL) for an additional 12 or 24 h, and protein lysates were extracted in radioimmunoprecipitation (RIPA) lysis buffer containing 4% protease inhibitor, 4% phosphorylase inhibitor, and 1% PMSF. The protein concentration was determined using the BCA assay. Equal amounts of protein lysate were separated by sodium dodecyl sulfate-polyacrylamide gel electrophoresis (SDS-PAGE). The proteins were transferred to polyvinylidene fluoride membranes (Bio-Rad Laboratories), which were blocked with 5% fat-free milk in PBS-Tris buffer for 2 h and then incubated overnight at 4°C with primary antibodies. TBST was used to wash away excess antibodies. The membranes were then incubated with the secondary antibody for 2 h at room temperature. Next, TBST was used to wash away excess secondary antibodies. Protein detection was performed using ECL reagent (GE Healthcare, USA). The results are reported as ratios of expression relative to that of β-actin.

### qRT-PCR

MC3T3-E1 cells were treated as described above, and total RNA was extracted using Trizol reagent (Sigma, USA). cDNA was synthesized using Roche reagent (4896866001; Transcriptor cDNA Synthesis Kit 1; USA). Real-time PCR analysis was performed using a C1000 thermocycler and an ABI7500 real-time PCR system (Applied Biosystems, USA) with a Roche Fluorometric Quantitation Kit (04913850001, FastStart Universal SYBR Green Master (ROX)).

The amplified products were measured using amplification curve analysis. All data were analyzed using the 2^-ΔΔCT^ method and were normalized to the house-keeping gene β-actin. The primer pairs are presented in Table 1.

**Table 1.** Primer pairs used in the study.

	Forward	Reverse
β-actin	5′-GGCTGTATTCCCCTCCATCG-3′	5′-CCAGTTGGTAACAATGCCATGT-3
RIPK3	5′-GAACTGAAGAAGCTGGAGTTTGTG-3′	5′-ATCTTGACTGCTACATCATGGTTCC-3′
MLKL	5′-AATTGTACTCTGGGAAATTGCCA-3′	5′-CTCCAAGATTCCGTCCACAG-3′
Caspase 3	5′-TGGTGATGAAGGGGTCATTTATG-3′	5′-TCGGCTTTCCAGTCA-GACTC-3′

### Statistical analysis

Data are reported as means±SD from at least three independent technical replicates. Data were analyzed using SPSS 22.0 software (IBM, USA).

## Results

### Nec-1 restored MC3T3-E1 cell viability reduced by TNF-α plus Z-IETD-FMK

**Figure 1.**
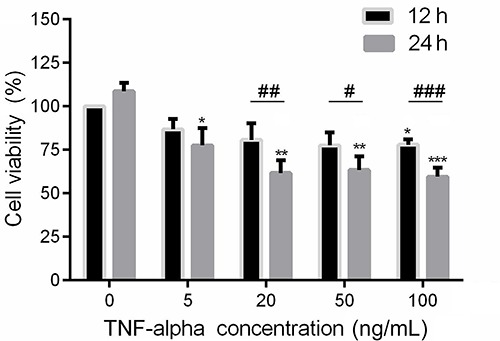
Cell viability of MC3T3-E1 cells was measured using the CCK8 assay. Data are reported as means±SD of five independent experiments. Data on the y-axis indicate cell viability relative to the group of cells that were incubated for 12 and 24 h without tumor necrosis factor-α (TNF-α). *P<0.05, **P<0.01, ***P<0.001 compared to control; ^#^P<0.05 between the same concentration at different time-points (Student's *t*-test).

When MC3T3-E1 cells were treated with TNF-α, their viability decreased as the TNF-α concentration increased. When cells were treated with a single concentration of TNF-α, their viability decreased over time. Treatment with 20 ng/mL TNF-α significantly reduced cell viability. Taken together, these results demonstrated that TNF-α inhibited MC3T3-E1 cell viability in a dose- and time-dependent manner (Figure 1).

**Figure 2.**
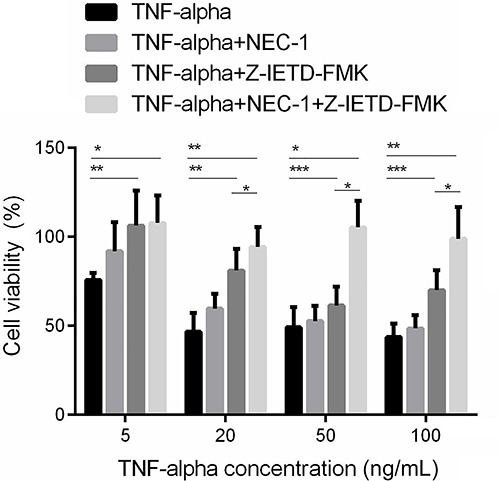
The viability of MC3T3-E1 cells was measured by the CCK-8 assay. The cells were treated with Z-IETD-FMK (40 µM), Nec-1 (50 µM), or Z-IETD-FMK (40 µM) combined with Nec-1 (50 µM) for 1 h and then co-cultured with tumor necrosis factor-α (TNF-α) for 24 h. The control group was treated with TNF-α without any inhibitor. Data on the y-axis indicate cell viability relative to the group of cells without any treatment. *P<0.05, **P<0.01, ***P<0.001 compared with control treatment (ANOVA).

When cells were treated with 20 ng/mL TNF-α, Nec-1, and Z-IETD-FMK, the cell viability trends were different. Nec-1 did not significantly increase cell viability, while the caspase-8 inhibitor Z-IETD significantly increased viability (P<0.05). These results suggested that the cell death induced by treatment with TNF-α was primarily mediated by apoptosis, not necroptosis. However, when the cells were treated with TNF-α plus Z-IETD, Nec-1 significantly enhanced cell viability compared with Z-IETD treatment alone (Figure 2, P<0.05). This demonstrated that inhibiting apoptosis induced necroptosis, and that Nec-1 protected the cells from TNF-α plus Z-IETD-induced death.

### Nec-1 reversed cell population shift caused by TNF-α plus Z-IETD-FMK

**Figure 3.**
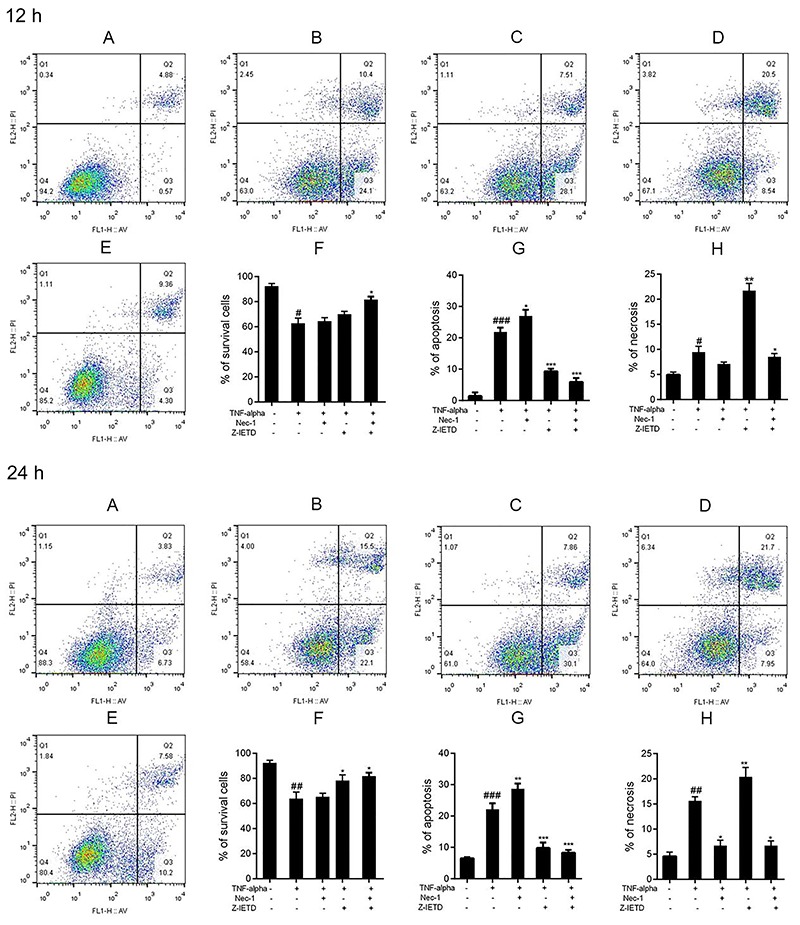
Representative graphs of cell death (12 and 24 h) by flow cytometry analysis after Annexin-V/PI dual staining. Control *A*: untreated cells; *B*: tumor necrosis factor-α (TNF-α) alone; *C*: TNF-α + Nec-1; *D*: TNF-α + Z-IETD-FMK; *E*: TNF-α + Nec-1 + Z-IETD-FMK. *F*: Cell survival ratio; *G*: Apoptosis; *H*: Cell necrosis ratio. We used the population of A-P- for cell survival, A+P- for apoptosis and A+P+ for necrosis. Data are reported as means±SD of three independent experiments. ^#^P<0.05, ^##^P<0.01, ^###^P<0.001 *vs* control treatment; *P<0.05, **P<0.01, ***P<0.001 *vs* TNF-α (ANOVA).

Flow cytometric analysis showed that treatment with TNF-α resulted in a rapid increase in the percentage of AV+PI− and AV+PI+ cells. To distinguish between late apoptotic cells and necrotic cells in the AV+PI+ population, cell death was assessed at two time-points (12 and 24 h). As shown in Figure 3B (12 h), treatment with TNF-α increased the percentage of AV+PI− and AV+PI+ cells. After an additional 12 h of treatment, as shown in Figure 3B (24h), there was a further increase in the percentage of AV+PI+ cells. Thus, we concluded that the AV+PI+ cells were late apoptotic cells. Similarly, when the cells were treated with Nec-1 (a specific inhibitor of necroptosis) to block RIPK1, TNF-α-induced cell death was not inhibited (Figure 3C (12, 24 h)), while treatment with the caspase-8 inhibitor Z-IETD improved cell survival (Figure 3D (12, 24 h)). These results suggested that cell death occurred primarily through apoptosis, not necroptosis. When the cells were treated with Z-IETD alone in the presence of TNF-α, there was a shift in the cell population from AV+PI− to AV+PI+ (Figure 3D (12, 24 h)). Moreover, there was no significant change in the percentage of AV+PI+ cells at 24 h compared with at 12 h (Figure 3D). This suggested that the inhibition of apoptosis increased the frequency of necroptosis in the presence of TNF-α. Treatment with Nec-1 reversed this effect (Figure 3E (12, 24 h)), which suggests that typical necroptosis was taking place.

### Z-IETD-FMK plus Nec-1 significantly decreased LDH release

**Figure 4.**
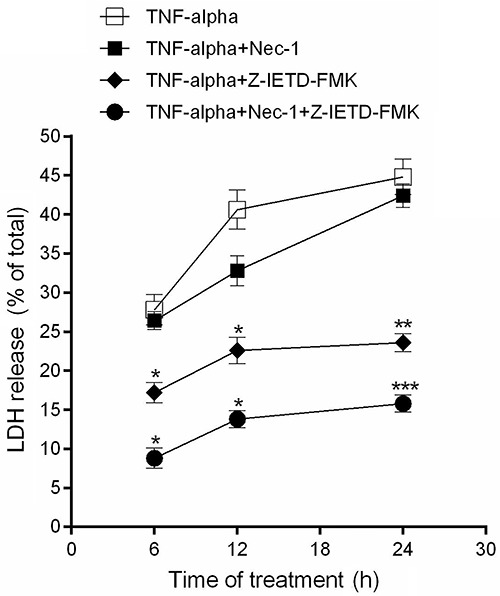
Lactate dehydrogenase (LDH) release assay. Cells were treated with Z-IETD-FMK (40 µM), Nec-1 (50 µM), or Z-IETD-FMK (40 µM) combined with Nec-1 (50 µM) for one hour, followed by co-culture with tumor necrosis factor-α (TNF-α) (20 ng/mL) for 6, 12, and 24 h. Data are reported as means±SD from five independent experiments. *P<0.05, **P<0.01, ***P<0.001 *vs* TNF-α (Student's *t*-test).

At the first time-point tested (6 h), treatment with TNF-α resulted in a significant increase in LDH release. When the cells were treated with Nec-1, LDH release did not decrease compared with treatment with TNF-α alone, while treatment with Z-IETD-FMK alone significantly decreased LDH release (P<0.05). Treatment with Z-IETD-FMK plus Nec-1 resulted in a significant decrease in LDH release over time compared with treatment with TNF-α (Figure 4, P<0.001), suggesting that Z-IETD-FMK plus Nec-1 protected the cells from dying.

### TNF-α plus Z-IETD-FMK increased RIPK3 and MLKL phosphorylation

**Figure 5.**
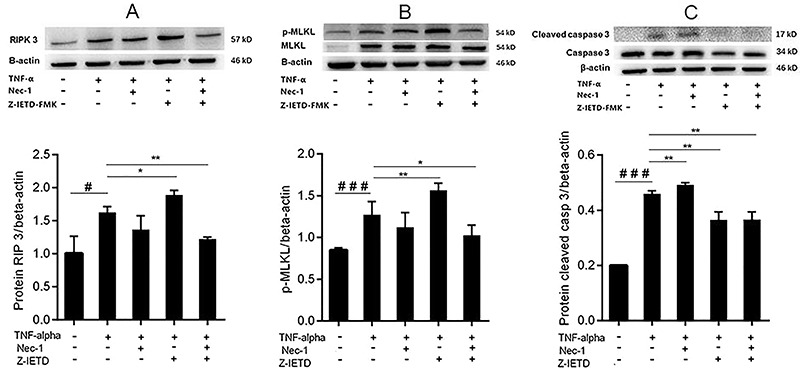
Representative western blot graphs showing the effects of tumor necrosis factor-α (TNF-α) on the protein expression levels of Specific marker proteins receptor interacting protein kinase (RIPK3) (*A*), mixed lineage kinase domain-like protein (MLKL) and p-MLKL (*B*), caspase 3 and cleaved caspase 3 (*C*), and β-actin in MC3T3-E1 cells. Data are reported as means±SD from three independent experiments. ^#^P<0.05, ^###^P<0.001 *vs* Control, *P<0.05, **P<0.01 *vs* TNF-α (Student’s *t*-test).

Treatment with TNF-α alone significantly increased the expression of cleaved caspase 3 (Figure 5C). When the cells were treated with Z-IETD-FMK, the level of cleaved caspase 3 decreased significantly (P<0.001), while the expression of RIPK3 and MLKL phosphorylation, key proteins in the necroptosis pathway, increased significantly (Figure 5A and B, P<0.05). When the cells were treated with Nec-1 alone, the level of cleaved caspase 3 increased (Figure 5C, P<0.01). Moreover, treatment with Nec-1 and Z-IETD-FMK resulted in a significant decrease in RIPK3, p-MLKL, and cleaved caspase 3 expression levels. The results indicated TNF-α plus Z-IETD-FMK induced necroptosis, while TNF-α plus Nec-1 induced apoptosis.

### mRNAs were not consistent with corresponding proteins

**Figure 6.**
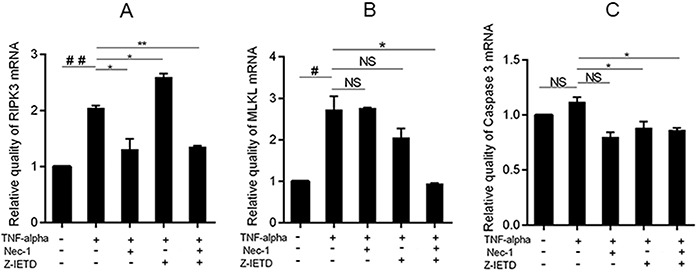
RT-PCR was conducted to measure mRNA levels of receptor interacting protein kinase (RIPK3) (*A*), mixed lineage kinase domain-like (MLKL) (*B*), and caspase 3 (*C*) in MC3T3-E1 cells. Data from the treated groups were normalized to β-actin. Cells without any treatment served as the control group. Data are reported as means±SD from three independent experiments. ^#^P<0.05, ^##^P<0.01, *vs* Control, *P<0.05, **P<0.01 *vs* tumor necrosis factor-α (TNF-α) (Student's *t*-test). NS: not significant.

Treatment with TNF-α significantly increased RIPK3 (Figure 6A, P<0.01) and MLKL (Figure 6B, P<0.05) mRNA expression levels. Treatment with Nec-1 alone decreased the expression of RIPK3 mRNA, but did not have any effect on MLKL and caspase 3 mRNA expression levels. Treatment with Z-IETD-FMK increased RIPK3 mRNA expression and decreased caspase 3 mRNA expression (P<0.05), but had no effect on MLKL. When the cells were treated with Nec-1 and Z-IETD-FMK, RIPK3, MLKL, and caspase 3 mRNA levels decreased significantly (P<0.05). The changes in RIPK3, MLKL and caspase 3 mRNA levels were not consistent with the changes in the corresponding protein expression levels.

## Discussion

In this study, we demonstrated for the first time that necroptosis was involved in the regulated cell death of mouse osteoblasts treated with TNF-α. This conclusion is supported by several independent lines of evidence. First, TNF-α plus Z-IETD triggered necroptosis in MC3T3-E1 cells. Second, Nec-1 inhibited a core element of the necroptosis pathway, RIPK1, and down-regulated the expression of RIPK3 and MLKL phosphorylation. Third, treatment with Nec-1 prevented necroptosis when the cells were treated with TNF-α plus Z-IETD, as shown by the increased cell viability and reduced percentage of necrotic cells.

In addition, cell viability decreased as the TNF-α concentration increased, and in cells treated with the same concentration of TNF-α, cell viability decreased over time. Treatment with 20 ng/mL TNF-α significantly inhibited cell viability. Taken together, these results demonstrated that TNF-α induced MC3T3-E1 cell death in a dose- and time-dependent manner. This phenomenon has also been observed by Zheng, et al. (10) in osteoblast cells.

Treatment with TNF-α plus Nec-1 did not reduce cell viability and increase LDH release with significance. However, the population of A+PI− cells and the expression of cleaved caspase 3, the specific protein in apoptosis pathway, increased significantly, suggesting that Nec-1 may induce apoptosis. TNF-α plus Z-IETD-FMK significantly increased the expression of protein RIPK3 and MLKL phosphorylation, indicating TNF-α plus Z-IETD-FMK induced necroptosis. These results may confirm Galluzzi et al. findings, which suggest that apoptosis and necroptosis may occur simultaneously, and that necroptosis can even be converted to apoptosis under specific conditions (11).

Differently from other types of cells, Z-IETD plus TNF-α decreased LDH release in the osteoblast cell line. The result may indicate that TNF-α induced osteoblast cell death mostly in the manner of apoptosis and subsequent late apoptosis, whereas necroptosis induced by Z-IETD plus TNF-α only played a relatively limited part. Therefore, Z-IETD plus TNF-α protected from LDH release more than increased LDH release by inducing necroptosis.

Simultaneously blocking necroptosis and apoptosis provided greater protection to cells treated with TNF-α than blocking either cell death route individually. Treatment with Nec-1 and Z-IETD-FMK increased cell viability and cell survival and decreased LDH release more effectively than treatment with Nec-1 or Z-IETD-FMK alone. This is because Nec-1 inhibits necroptosis, which is induced by Z-IETD-FMK plus TNF-α. Thus, when TNF-α-induced apoptosis is blocked by Z-IETD-FMK, necroptosis still occurs.

Some of our results are unclear and require further study. For example, the changes in RIPK3, MLKL, and caspase 3 mRNA levels were not consistent with the changes in the corresponding protein expression levels. This may be because we detected the active or functional forms of the proteins. Thus, future studies should explore the exact relationship between mRNA and nonfunctional protein expression levels.

Our findings may lead to the development of an effective strategy for the treatment of osteonecrosis after bone cement implantation. Osteonecrosis has been confirmed in vertebral bone tissue sections after PMMA (polymethyl methacrylate) implantation (12). We previously showed that TNF-α mRNA levels increase significantly at the cement-bone interface after injection of PMMA into the vertebral body of rats. An earlier study showed that TNF-α can induce osteoblast apoptosis (13) and our study confirmed this conclusion. In addition, we verified the protective effect of Z-IETD-FMK and Nec-1 on osteoblasts treated with TNF-α. Therefore, our findings may open new venues for the clinical treatment of bone necrosis caused by bone cement implantation. In conclusion, TNF-α induced preferentially apoptosis in osteoblast cell line and necroptosis played a decisive role when TNF-α-induced death was inhibited by the inhibitor of apoptosis. Combined treatment with Nec-1 and Z-IETD-FMK protected mouse osteoblasts from death induced by TNF-α.
